# Variation in airborne fungal spore concentrations among five monitoring locations in a desert urban environment

**DOI:** 10.1007/s10661-018-7008-5

**Published:** 2018-10-18

**Authors:** Tanviben Y. Patel, Mark Buttner, David Rivas, Chad Cross, Dennis A. Bazylinski, Joram Seggev

**Affiliations:** 1 Department of Environmental and Occupational Health, School of Community Health Sciences, University of Nevada, Las Vegas, 4505 S. Maryland Pkwy, Box 454009, Las Vegas, NV 89154 USA; 2 School of Medicine and School of Community Health Sciences, University of Nevada, Las Vegas, Las Vegas, NV USA; 3 School of Life Sciences, University of Nevada, Las Vegas, Las Vegas, NV USA

**Keywords:** Fungal spore, Airborne, Allergen, Outdoor air quality

## Abstract

Fungal spores are biological particles that are ubiquitous in the outdoor air. Spores of several very common fungal species are known allergens, with the potential to cause respiratory illnesses by exacerbating asthma and allergic rhinitis. The National Allergy Bureau typically has one monitoring station established per city to determine fungal spore counts for an entire metropolitan area. However, variations in fungal spore concentrations could occur among different locations. The objective of this study was to measure and compare airborne fungal spore concentrations in five locations in Las Vegas for the year 2015 to determine if there are differences among microenvironments in the city. Twenty-four-hour or 7-day air samples were collected from five sites across the Las Vegas Valley. Samples were analyzed with a light microscope for fungal spores and counts were converted to concentrations of spores per volume of air. Mixed-model methods were used to evaluate mean differences. Results showed that smuts (basidiomycetes) were the dominant spore type for all five sites during the spring season. *Cladosporium* species were responsible for the second most dominant spore type with the highest concentrations occurring during the summer and fall months. Results obtained from the five stations established in Las Vegas show that there are important variations among the sites regarding fungal spore concentrations. The data suggest that more sites and additional monitoring of outdoor allergens are needed to provide information necessary to inform the community of outdoor air quality conditions and their potential effects on public health. This study presents new outdoor fungal spore data for the southwest region of the USA, focused in the Las Vegas Valley.

## Introduction

Fungal spores are biological particles ubiquitous in the outdoor air. They typically range in size from 2 to 50 μm in diameter, with most allergenic spores in the respirable size range of 3 to 10 μm (Filippo et al. 2013; Yang and Johanning 2007). Dispersal of the spores is dependent on various factors, including airflow, water content, animal activity, and the movement of hosts (Lee et al. 2010). Meteorological and climate conditions, such as temperature and relative humidity, influence the concentrations of spores in the air (Ponce-Caballero et al. 2013). For example, dry air spore fungi can disperse long distances under low relative humidity and high wind speed (Kasprzyk and Worek 2006). Wet weather spores tend to disperse during and after rainfall (Khattab and Levetin 2008). Typically, fungal spore concentrations increase during spring and reach high levels in the summer (Haas et al. 2014). During high concentrations of fungal spores in the outdoor air, there are relatively higher concentrations found indoors because outdoor spores are transported indoors through air conditioning systems, windows, and doors (Ponce-Caballero et al. 2013).

Spores of several very common fungal species are known allergens, including those from *Cladosporium*, *Penicillium*, *Aspergillus*, various basidiomycetes, and *Alternaria* (Stetzenbach and Krauter 2016). In a previous study conducted in Spain and Brazil, ascomycete spores were the most prevalent in the urban environment, with *Cladosporium* accounting for the major taxa (Núñez et al. 2016). In addition, exposure to outdoor fungal spores has been shown to be a potential human health risk because of toxic substances called mycotoxins, produced by certain species (Fernández-Rodríguez et al. 2014). Fungal spores have the potential to cause respiratory illnesses by exacerbating asthma and allergic rhinitis (Stetzenbach and Krauter 2016). Fungal spores require only a small amount of moisture and oxygen, thus making the Mojave Desert a suitable environment for their survival (Levetin et al. 2012).

Typically, the National Allergy Bureau (NAB) uses one or two monitoring stations per city to obtain the airborne fungal spore counts for an entire metropolitan area (National Allergy Bureau 2017). However, variations in fungal spore concentrations can occur among different microenvironments, resulting in differences in human exposure to allergenic fungal spores. The objective of this study was to measure and compare airborne fungal spore concentrations in five locations in Las Vegas to determine whether differences occur between microenvironments within the city.

## Methods

### Air sample collection and analysis

Air samples were collected from five sites in the Las Vegas Valley using a Burkard spore trap (Burkard Manufacturing Company, Rickmansworth, Hertfordshire, England) placed onto rooftops. Samples were collected from April 7, 2015, to April 6, 2016. Either 24-h or 7-day samples were collected. For 24-h samples, a 3″ × 1″ plain glass microscope slide was coated with a thin film of high vacuum grease (Dow Corning Corporation, Midland, Michigan) and inserted into the sampler. Airborne particles were impacted onto the slide for 24 h at an air flow rate of 10 l per minute (Levetin et al. 2000). The samples were collected daily, and each slide was placed onto a slide warmer for 10 min (Electron Microscopy Sciences® Hatfield, Pennsylvania). A coverslip was then applied with a few drops of glycerin jelly stained with basic fuchsin (Levetin et al. 2000). For 7-day samples, a strip of Melinex® tape (New Berlin, Wisconsin) was affixed to the 7-day sampler drum and coated with a thin film of Dow Corning® grease. The sampler drums were changed weekly, and the tapes were cut into 48-mm segments, representing 24-h increments. The cut sections were affixed to a microscope slide with a 10% Gelvatol solution (Robert E. Esch, Lenoir, NC) and allowed to dry for 10 min. Coverslips were then applied with a few drops of glycerin jelly stained with basic fuchsin. The prepared slides were analyzed with a light microscope at a total magnification of × 1000 for fungal spores. A single longitudinal traverse was counted according to NAB protocols (Levetin et al. 2000). Fungal spores were identified, if possible, and counts were converted into airborne concentrations and expressed as fungal spores/m^3^ (Khattab and Levetin 2008).

### Study site

**Fig. 1:**
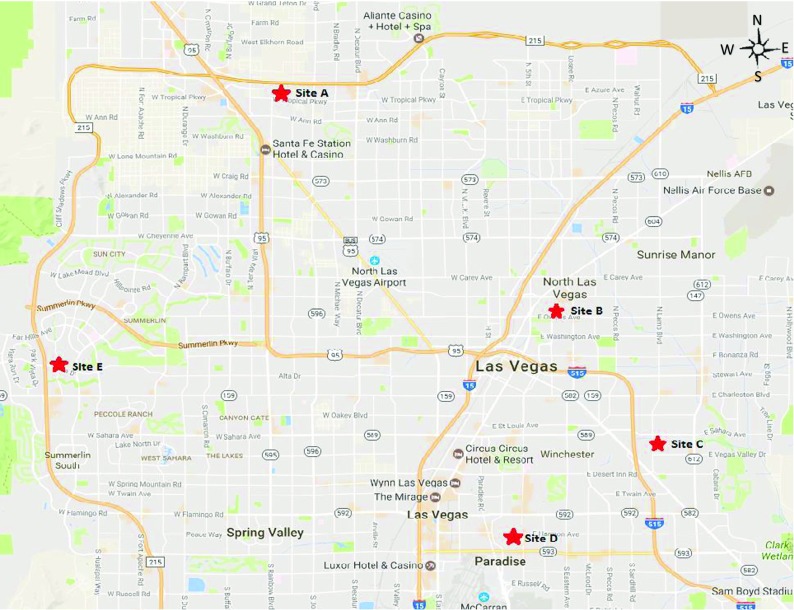
Map of airborne pollen collection sites in Las Vegas, NV (North ↑)

Five monitoring locations were established in Las Vegas (Fig. 1). Sites A, C, and E are located in newer developed areas in the city close to housing and small roads. Sites B and D are located in densely populated areas, in older parts of the city near busy roads. Samplers were mounted onto single-story rooftop structures for sites A, B, C, and E. Site D sampler was mounted on the roof of a three-story building. Sampling heights of the samplers were determined based on guidelines provided by the National Allergy Bureau that specify building heights of one to three stories (NAB training materials) and the availability of the location to accommodate a monitoring site.

In the Las Vegas Valley, the average altitude ranges from 680 to 701 m above sea level. Vegetation in the valley is dominated by desert urban landscaping featuring hardy, low water use plants. The temperatures range from a monthly average high of 104 °F to an average low of 38 °F (Table 1). The relative humidity ranges from 17 to 30% during the spring season and the driest days are typically in June, with a relative humidity of 13%. The average precipitation is 101 to 127 mm per year. The typical wind speed ranges from 0 to 4 m per second (mps), with average wind gusts of 12 mps.

**Table 1:** Meteorological data for Las Vegas, NV, in 2015 and 2016 (source: timeanddate.com and wunderground.com)

Year	Month	Mean high and low temperature (°C)	Mean relative humidity (%)	Total precipitation (mm)	Mean wind speed/gusts (mps)
2015	April	27/13	17	6.6	5/11
	May	29/18	25	6.1	4/10
	June	40/27	13	0.0	5/11
	July	38/27	21	4.8	4/11
	August	40/28	21	17.3	2/9
	September	37/24	22	0.5	3/9
	October	29/18	36	29.5	3/10
	November	18/7	31	6.1	3/12
	December	13/3	35	0.3	3/11
2016	January	14/4	46	11.7	2/11
	February	21/8	29	2.3	3/12
	March	24/12	24	0.0	4/10
	April	26/14	30	57.4	4/11

### Statistical analysis

A Shapiro-Wilk test and observation of skewness and kurtosis measures were used to determine if the data were normally distributed. For the analyses that followed, all data were log-transformed prior to analysis to meet the distributional assumptions of the modeling approach. A mixed-model analysis was used to assess potential differences among locations and months while treating measurements as repeated factors. The mixed-model approach allowed for the inclusion of both location and time, and additionally provided a means to account for an auto-regressive time lag in the data, which was important owing to the temporal nature of the data collection. A planned post hoc analysis based on marginal mean differences was used to determine differences among locations and months when the overall model suggested differences in main effects. This method is more robust and accounts for repeated methods compared to the two-way ANOVA. IBM SPSS software version 24 was used to analyze the data.

## Results

### Total pollen

**Fig. 2:**
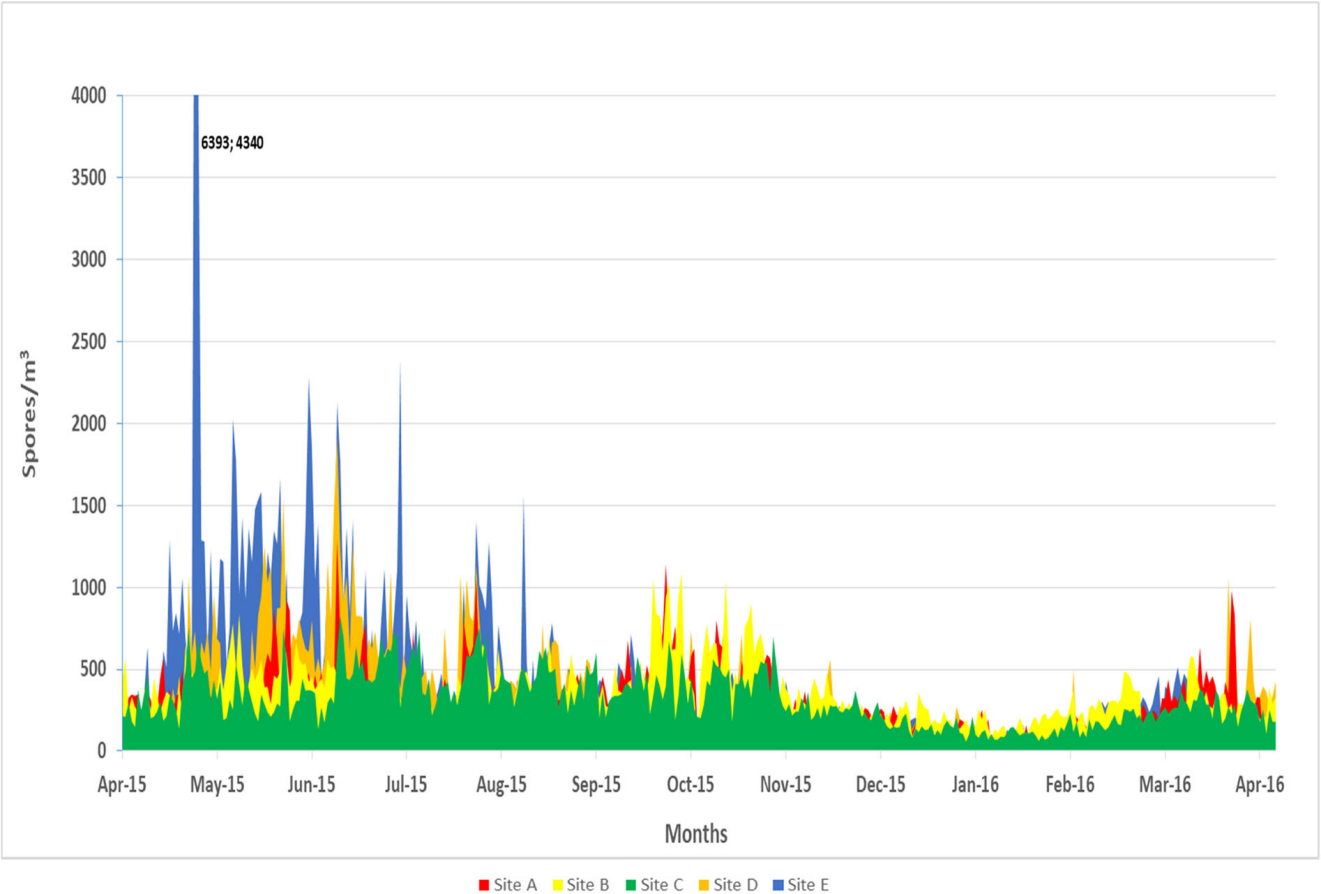
Total fungal spore counts at five locations in Las Vegas from April 2015 to April 2016 (spores/m^3^)

The fungal reproductive season typically starts at the beginning of the spring and peaks in the summer in the Las Vegas Valley. Site E had the greatest total fungal spore concentration (6393 spores/m^3^) in May 2015 and had the highest mean total fungal spore concentration (488 spores/m^3^) compared to sites A (329 spores/m^3^), B (366 spores/m^3^), C (318 spores/m^3^), and D (373 spores/m^3^). Sites A and B had the highest peaks during the fall months of September to November (Fig. 2).

**Fig. 3:**
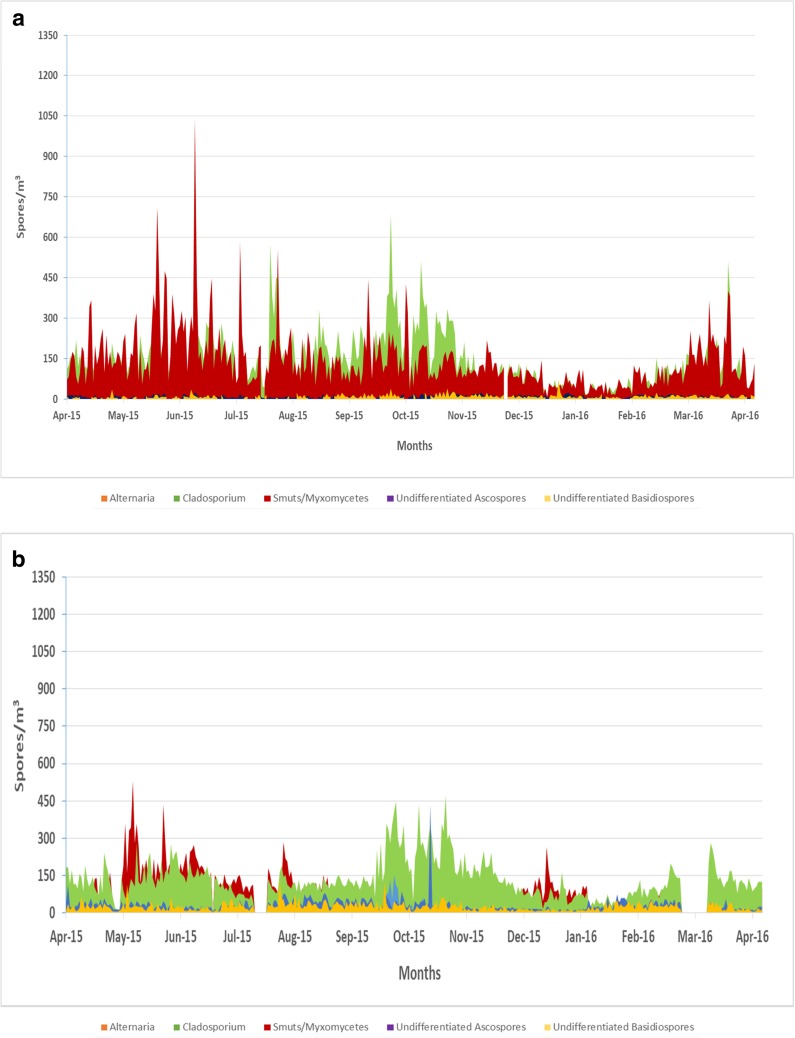

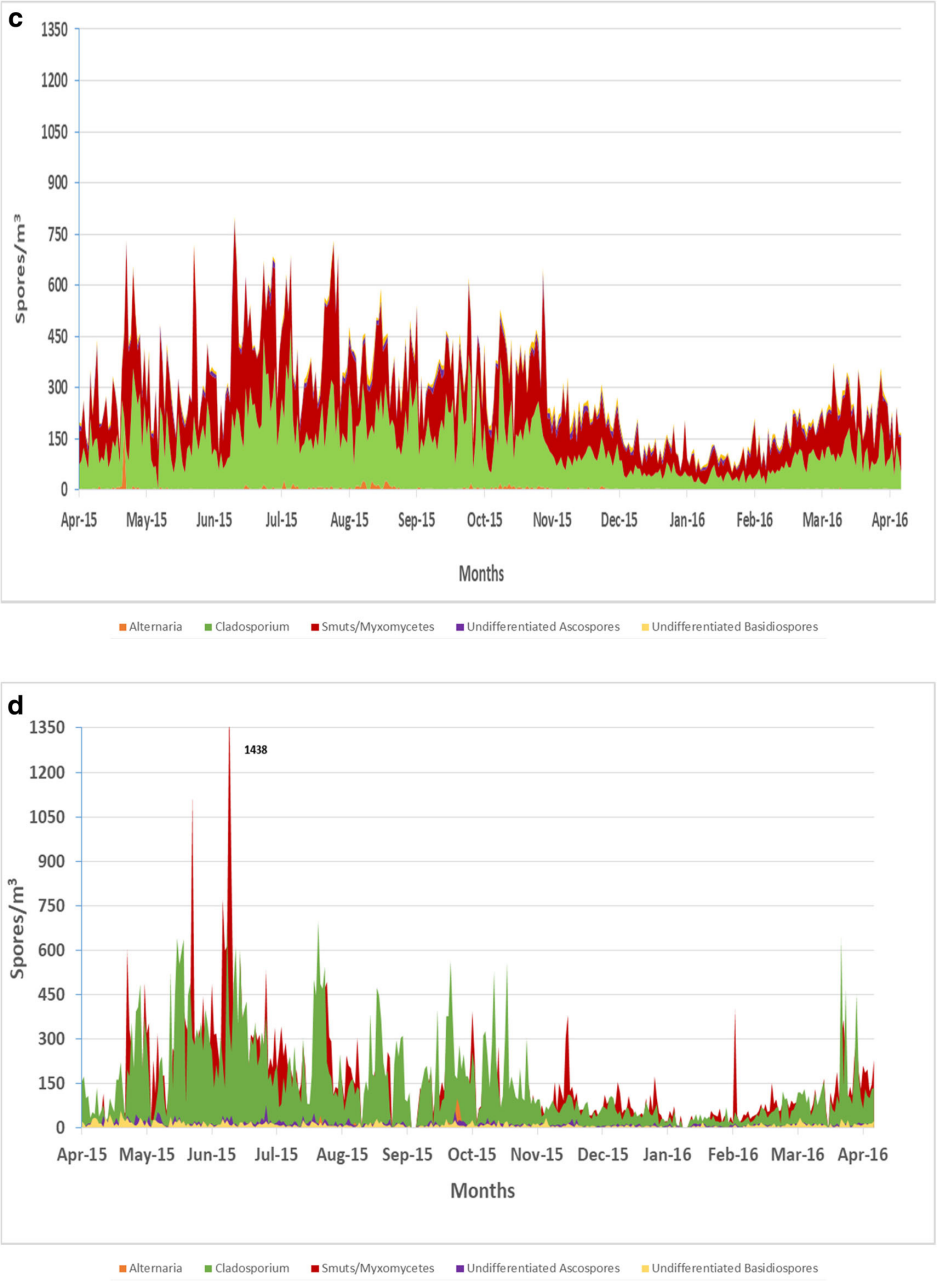

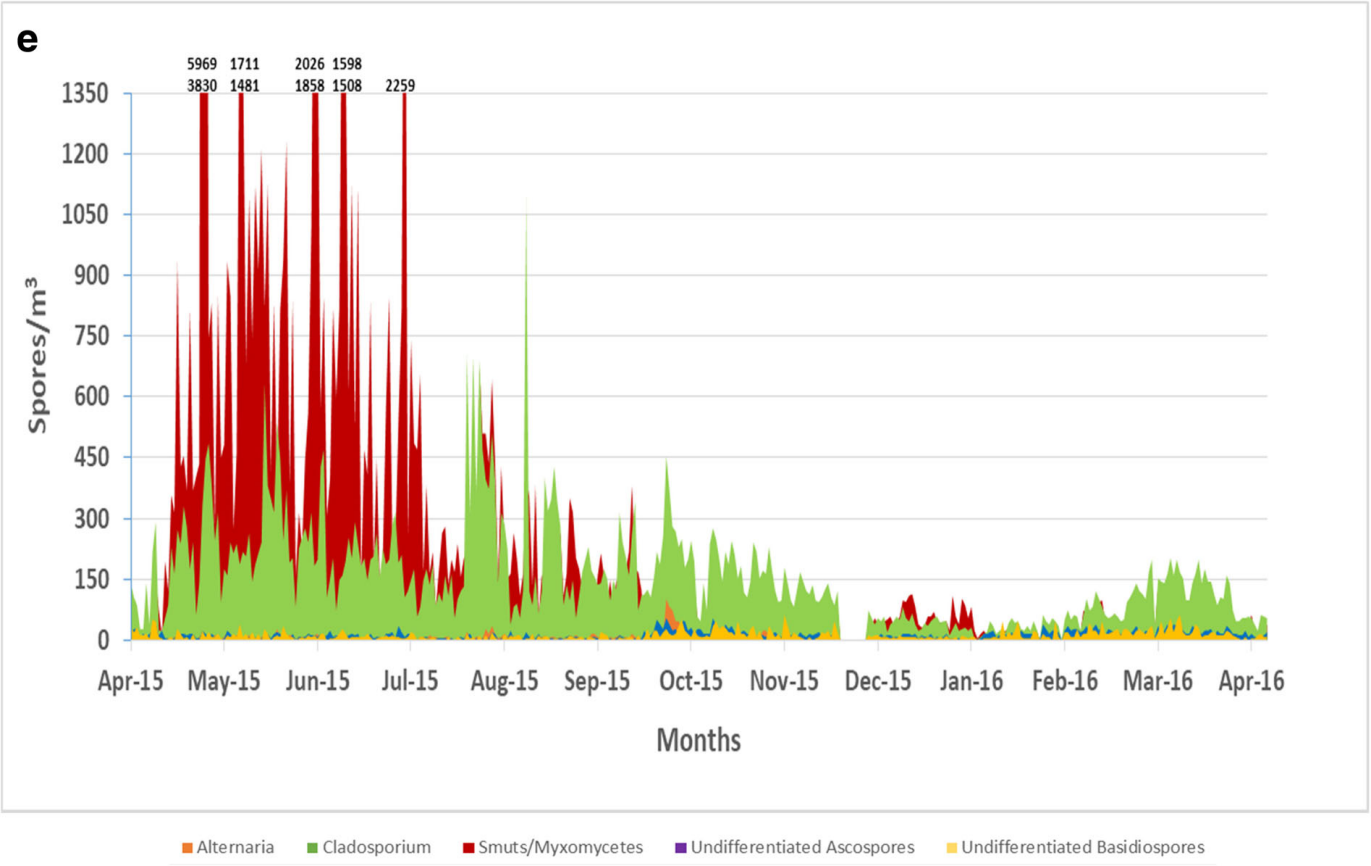
**a** Variation in airborne fungal spores for site A across the Las Vegas Valley (spores/m^3^). **b** Variation in airborne fungal spores for site B across the Las Vegas Valley (spores/m^3^). **c** Variation in airborne fungal spores for site C across the Las Vegas Valley (spores/m^3^). **d** Variation in airborne fungal spores for site D across the Las Vegas Valley (spores/m^3^). **e** Variation in airborne fungal spores for site E across the Las Vegas Valley (spores/m^3^)

Smut spores were the dominant spore type for all five sites during the spring season (Fig. 3a–e). The highest concentration of smut spore was at site E (5,969 spores/m^3^) in May (Figs. 2 and 3e). Smut spore concentrations were the lowest at site B when compared to all of the other sites (*p* < 0.05). *Cladosporium* species were responsible for the second dominant spore type with the highest concentrations occurring during the summer and fall months. Sites D (mean = 162 spores/m^3^) and E (mean = 158 spores/m^3^) had the highest concentrations of *Cladosporium* spores annually (Fig. 3d, e). Total *Cladosporium* spore concentrations showed no significant differences among the five sites.

Airborne fungal spore concentrations were significantly different among locations (Table 2). The greatest mean differences for *Alternaria* spores were between sites B and C. Ascospores had the greatest mean difference between sites B and A. Basidiospores also had the greatest difference between sites B and A. The total fungal spore concentration had the largest mean difference between sites B versus C and sites E versus C.

**Table 2:** Comparison of airborne fungal spore concentrations in Las Vegas (only significant differences are shown)

Fungal type	Location (log mean)	Location (log mean)	Log mean difference	*p* value	95% confidence interval	
					Lower bound	Upper bound
*Alternaria*	A (0.419)	C (0.344)	0.075	0.044	0.002	0.149
	B (0.736)	A (0.419)	0.316	< 0.001	0.242	0.391
	B (0.736)	C (0.344)	0.392	< 0.001	0.317	0.466
	B (0.736)	D (0.422)	0.314	< 0.001	0.239	0.389
	B (0.736)	E (0.516)	0.220	< 0.001	0.144	0.295
	D (0.422)	C (0.344)	0.078	0.039	0.004	0.151
	E (0.516)	A (0.419)	0.097	0.011	0.023	0.171
	E (0.516)	C (0.344)	0.172	< 0.001	0.098	0.246
	E (0.516)	D (0.422)	0.095	0.013	0.020	0.169
*Cladosporium*	No differences among the 5 sites					
*Smuts*	A (2.018)	B (1.881)	0.137	0.002	0.051	0.224
	C (2.022)	B (1.881)	0.141	0.001	0.055	0.228
	D (1.972)	B (1.881)	0.092	0.038	0.005	0.178
	E (2.025)	B (1.881)	0.153	0.001	0.066	0.239
*Ascospores undifferentiated*	B (1.386)	A (0.906)	0.480	< 0.001	0.417	0.543
	B (1.386)	C (0.992)	0.395	< 0.001	0.332	0.458
	B (1.386)	D (1.090)	0.296	< 0.001	0.233	0.359
	B (1.386)	E (1.034)	0.353	< 0.001	0.289	0.416
	C (0.992)	A (0.906)	0.086	0.007	0.023	0.148
	D (1.090)	A (0.906)	0.184	< 0.001	0.122	0.247
	D (1.090)	C (0.992)	0.099	0.002	0.036	0.161
	E (1.034)	A (0.906)	0.128	< 0.001	0.065	0.190
*Basidiospores undifferentiated*	B (1.289)	A (0.804)	0.486	< 0.001	0.421	0.551
	B (1.289)	C (0.920)	0.370	< 0.001	0.305	0.435
	B (1.289)	D (0.998)	0.292	< 0.001	0.227	0.357
	B (1.289)	E (0.958)	0.331	< 0.001	0.266	0.396
	C (0.920)	A (0.804)	0.116	< 0.001	0.052	0.180
	D (0.998)	A (0.804)	0.194	< 0.001	0.130	0.258
	D (0.998)	C (0.920)	0.078	0.017	0.014	0.142
	E (0.958)	A (0.804)	0.155	< 0.001	0.091	0.219
Total fungal spores	B (2.476)	A (2.410)	0.066	0.019	0.011	0.121
	B (2.476)	C (2.404)	0.072	0.011	0.017	0.128
	B (2.476)	D (2.408)	0.068	0.017	0.012	0.123
	E (2.475)	A (2.410)	0.065	0.020	0.011	0.120
	E (2.475)	C (2.404)	0.072	0.011	0.017	0.127
	E (2.475)	D (2.408)	0.067	0.017	0.012	0.122

*Alternaria* spores had significant differences in concentrations across at all sites, particularly site B, which had the largest mean differences from all other sites. Site E had significant mean differences among sites A, C, and D. Ascospores at site B had the largest differences in means versus site A, site C, site D, and site E. Basidiospores at site B also had the largest differences in means versus sites A, C, D, and E. The concentrations of total fungal spores showed large mean differences at sites B versus A, C, and D. There were also significant differences seen at sites E versus A, C, and D (Table 2).

## Discussion

Data are relatively sparse for outdoor airborne fungal spore concentrations in Las Vegas. In our study, the total concentrations of fungal spores showed variation among the five sites, specifically at site E, which had the highest concentrations. This site is located in a newer developed area in the city, and it is close to housing and small roads. The total fungal concentrations obtained in Las Vegas were two to five times greater than those obtained in a study that measured outdoor colony forming units (CFU) of fungal in the far western USA (Shelton et al. 2002). However, CFU only quantify a fraction of the total fungal counts seen in air samples. Our results were similar to a study in New Delhi where total fungal spore concentrations from slide counts showed site-to-site variations (Gupta et al. 1993). A study in New Orleans, LA, showed significant differences in daily fungal spore concentrations between flooded (66,167 spores/m^3^) and non-flooded (33,179 spores/m^3^) sites (Solomon et al. 2006), while a study in the state of West Bengal, India, had total fungal spore concentrations of approximately 2500 spores/m^3^ at one of their five collection sites during the spring months (Adhikari et al. 2004). In contrast, the peak concentration seen for total fungal spores at site E in Las Vegas was 6393 spores/m^3^.

*Cladosporium* spore concentrations showed no significant differences among the five sites in Las Vegas. These results were consistent with findings from a study conducted in 2005, in Dublin, Ireland, that showed only marginal differences in *Cladosporium* concentrations among the four sites and no statistical difference (O’Gorman and Fuller 2008). One explanation for the similarities between sites is the observation that spores from *Cladosporium* species are the most commonly found outdoor airborne fungal spore in various regions of the world (Shelton et al. 2002). However, our results showed variations in concentrations of *Alternaria* spores at the five locations in Las Vegas. Our results were consistent with studies done in Saudi Arabia, in which there were differences in *Alternaria* spore concentrations between two different centers sampled (Hasnain et al. 1998). A study in Porto, Portugal, identified higher concentrations of spores of *Alternaria* in the urban environment compared to other spores (Oliveira et al. 2010).

Smut spores (basidiomycetes) were the dominant spores for all five sites during the spring season. The highest peak of smuts was 5969 spores/m^3^. The study in New Delhi conducted in 1989–1990 collected from five sites around the city showed significant variations between the sites for smuts and total airborne fungal spore concentrations. The highest peak for smuts spores in New Delhi was 3000 spores/m^3^ in March, similar to our results (Gupta et al. 1993). A study in Mexico City showed variation among two sampling sites for basidiomycete spores, which included smuts during the dry seasons (October–May) (Calderon et al. 1995).

While urban fungal spore concentrations have been measured, variability of data within urban microenvironments has not been reported, to our knowledge. The results of this study indicated that there were differences among the five sites, especially between site B and site D. Site B showed the most variation for ascospores, basidiospores, and total fungal spores when compared to the other sites. Site E displayed the greatest differences when compared to sites A and C for *Alternaria*, Ascospores, Basidiospores, and total fungal spores. There was also a difference between site E and site B for smut concentrations. The reasons for the observed differences in airborne fungal spore concentrations are unknown. There were also no observed relationships between precipitation amount and total fungal spore concentrations.

The primary limitation of this study is the availability of data for a limited timeframe. One year of data provides interesting information about the seasonal variation of spores, but additional years of data would provide a more complete temporal picture. Additionally, five sites may not provide a complete geographic picture of airborne fungal spore concentrations around the Las Vegas Valley, particularly if microenvironments differ markedly across geography.

Typically, there are one to two NAB stations per metropolitan area to quantify the concentrations of fungal spores for the area. The results obtained from the five stations established in Las Vegas have shown that there are important variations among the five sites. Our study suggests that more sites and additional monitoring of outdoor allergens are needed to provide information necessary to inform the community of outdoor air quality conditions and their effects on public health. This study presented new outdoor fungal spore data for the southwest region of the USA, focused in the Las Vegas Valley. The results demonstrated variability among the fungal spore concentrations at different locations in the valley and provide a baseline for future research in outdoor air quality in the Mojave Desert.
